# Increasing burden of *E. coli* bacteraemia and changing epidemiology

**DOI:** 10.1186/1753-6561-5-S6-O3

**Published:** 2011-06-29

**Authors:** J Wilson, R Coello, A Jepson, E Brannigan, M Richards, S Hassall, A Holmes

**Affiliations:** 1 Infection prevention & control, Imperial College Healthcare NHS Trust, London, UK; 2 Microbiology, Imperial College Healthcare NHS Trust, London, UK

## Introduction / objectives

According to National surveillance in England *S. aureus* as a cause of bacteraemia has recently declined but that due to *E. coli has* increased by 33%. Mandatory reporting of E.coli is being introduced in 2011. At Imperial College Healthcare we investigated *E. coli* bacteraemia occurring between July 2008 and June 2010

## Methods

Microbiological records of patients with *E. coli* bacteraemia were linked with patient data to determine their characteristics, whether CA or HA, and if the *E. coli* produced ESBL. Blood cultures taken within 2 days of admission were defined as *CA*, 2 days or more after admission as HA and a new episode if greater than 2 weeks between positive blood cultures. *E. coli* ESBL was defined by resistance to cefpodoxime, or by resistance to ceftazidime or cefotaxime whilst remaining susceptible to cefoxitin. Where possible, the potential source of the bacteraemia was determined from the antibiotic profile of *E.coli* isolated concurrently from other specimens.

## Results

668 *E. coli* bacteraemia were detected (12% of all positive blood cultures), of which 67% were CA; 53% were female. For CA cases, the proportion of females (55%) was higher than males, whilst for HA cases, males accounted for more cases than females (47%). *E*, *coli* bacteraemia was more common in patients 65 years and over, accounting for 44% of CA and 49 % of HA cases. Of the 668, 110 (16%) produced ESBL, of which 61 (55%) were CA. Overall, 14% of CA and 22% of HA cases were ESBLs. The source was identified for 35% of cases; in 30% of these the urinary tract was responsible.

## Conclusion

*E.coli*, is an important cause of CA and HA bacteraemia with a significant proportion ESBL-strains. Mandatory reporting may facilitate understanding of the epidemiology and target prevention strategies.

## Disclosure of interest

None declared.

